# Effect of Stabilized nZVI Nanoparticles on the Reduction and Immobilization of Cr in Contaminated Soil: Column Experiment and Transport Modeling

**DOI:** 10.3390/nano14100862

**Published:** 2024-05-15

**Authors:** Hesham M. Ibrahim, Abdallah A. Al-Issa, Abdullah S. Al-Farraj, Abdulaziz G. Alghamdi, Ali M. Al-Turki

**Affiliations:** Department of Soil Science, College of Food and Agricultural Sciences, King Saud University, P.O. Box 2460, Riyadh 11451, Saudi Arabia; abdallah.allssa@narc.gov.jo (A.A.A.-I.); sfarraj@ksu.edu.sa (A.S.A.-F.); agghamdi@ksu.edu.sa (A.G.A.); aturki@ksu.edu.sa (A.M.A.-T.)

**Keywords:** nanosized zero-valent iron (nZVI), heavy metals, soil contamination, transport modeling

## Abstract

Batch and transport experiments were used to investigate the remediation of loamy sand soil contaminated with Cr(VI) using zero-valent iron nanoparticles (nZVI) stabilized by carboxymethylcellulose (CMC-nZVI). The effect of pH, ionic strength (IS), and flow rate on the removal efficiency of Cr(VI) were investigated under equilibrium (uniform transport) and non-equilibrium (two-site sorption) transport using the Hydrus-1D model. The overall removal efficiency ranged from 70 to over 90% based on the chemical characteristics of the CMC-nZVI suspension and the transport conditions. The concentration and pH of the CMC-nZVI suspension had the most significant effect on the removal efficiency and transport of Cr(VI) in the soil. The average removal efficiency of Cr(VI) was increased from 24.1 to 75.5% when the concentration of CMC-nZVI nanoparticles was increased from 10 to 250 mg L^−1^, mainly because of the increased total surface area at a larger particle concentration. Batch experiments showed that the removal efficiency of Cr(VI) was much larger under acidic conditions. The average removal efficiency of Cr(VI) reached 90.1 and 60.5% at pH 5 and 7, respectively. The two-site sorption model described (*r^2^* = 0.96–0.98) the transport of Cr(VI) in soil quite well as compared to the uniform transport model (*r^2^* = 0.81–0.98). The average retardation of Cr(VI) was 3.51 and 1.61 at pH 5 and 7, respectively, indicating earlier arrival for the breakthrough curves and a shorter time to reach maximum relative concentration at lower pH. The methodology presented in this study, combining column experiment and modeling transport using the Hydrus-1D model, successfully assessed the removal of Cr(VI) from polluted soils, offering innovative, cost-effective, and environmentally friendly remediation methodologies.

## 1. Introduction

Contamination with heavy metal pollutants has become a worldwide problem and a serious threat to the environment. Nowadays, there is a dramatic increase in global concern regarding the pollution of soil and water resources with heavy metal contaminants, mainly due to its detrimental impacts on human health and natural ecosystems [1,2,3]. Heavy metals contamination is usually related to several anthropogenic activities including industrial production, mining, and waste disposal sites [4]. Irrigation with low-quality water that is contaminated with heavy metals, due to the discharge of wastewater (e.g., mines and industrial wastewater) into different water resources, can significantly contribute to the accumulation of large amounts of heavy metals in the soil [5].

Several factors control the retention and transport of heavy metal contaminants in subsurface layers and groundwater including the type and concentration of heavy metal ions, the physiochemical soil properties, and climatic conditions. Biodegradation of heavy metals in soil is extremely difficult. Therefore, decontamination of polluted soil and water is usually carried out using highly reactive adsorptive materials, which reduce or adsorb heavy metals through different mechanisms [6,7]. In recent years, the interest in the application of zero-valent iron nanoparticles (nZVI) for the in situ remediation of contaminated soils has grown dramatically [8]. The high efficiency of nZVI in the remediation of heavy metal ions is highly attributed to their large surface area and potential mobility in the subsurface [9]. In addition, the strong reducing power of nZVI nanoparticles increases its ability to decontaminate heavy metals through reduction/precipitation mechanisms [10]. Due to their extremely small size and unique properties, nZVI nanoparticles have higher reactivity for a wide range of contaminants including heavy metal ions [11].

Chromium (Cr) compounds have been used in several industrial and manufacturing applications including the fabrication of steel alloys, dyeing, tanning, ceramics, and paints [12]. Excessive concentrations of Cr have been detected in the soils close to industrial sites of wood processing, leather tanning, and pigment production [13,14]. In Saudi Arabia, interest in pollution with heavy metal contaminants at disposal sites and in areas surrounding industrial regions has increased dramatically in recent years [15,16]. Studies have indicated the presence of several heavy metals (e.g., As, Cr, Pb, Co, Cd, Zn, and Cu) in the soils and groundwater resources in concentrations that ranged between acceptable to above threshold limits according to the standards of the World Health Organization (WHO) [17]. In this regard, several studies conducted in south Riyadh City have reported the presence of Cr in the soil in concentrations exceeding acceptable limits [14,18].

Several studies have investigated the in situ remediation of Cr(VI) using nZVI nanoparticles [19,20,21]. For example, the remediation efficiency of Cr(VI) reached 99% in soil contaminated by tannery wastes (Cr(VI) = 43.3 mg∙kg^−1^) when 5 g L^−1^ nZVI suspension was used for in situ remediation for a period of 40 days [20]. Similarly, Du et al. [12] reported a complete reduction in chromium ore processing residue containing 15.9 mg∙kg^−1^ Cr(VI) using 6% (*w*/*w*) nZVI nanoparticles. Despite the great potential for nZVI nanoparticles to remediate Cr contamination, the exposure of nZVI nanoparticles to the surrounding media, such as atmospheric oxygen and water, oxidizes the nZVI to iron oxides, which reduces the surface charge on the nZVI nanoparticles and highly decreases its reactivity. As dissolved oxygen and water interact with the active surfaces of nZVI nanoparticles, the particles are oxidized to iron oxides, which greatly reduces the reactivity of the particles [22]. Moreover, nZVI nanoparticles quickly agglomerate, forming aggregates in the microns size, hence limiting its transport in soil and reducing its efficiency in remediation processes [13].

To overcome these problems, nZVI nanoparticles are usually stabilized using polymer coatings to maintain reactivity and reduce aggregation [23]. For example, Yang et al. [24] assessed the stability of nZVI nanoparticles of nano-iron slurries using visual assessment of gravitational sedimentation and observed fast aggregation and settlement of the nZVI nanoparticles in bare as compared to stabilized nZVI nanoparticles. He and Zhao [25] applied a low concentration of carboxymethylcellulose (CMC) to stabilize Fe/pd bimetallic nanoparticles and reported greater mobility and reactivity when used for the dechlorination of trichloroethene. Shi et al. [19] investigated the kinetics of bentonite-supported nZVI nanoparticles for the removal of Cr(VI) from an aqueous solution. They found that B-nZVI could potentially be used as a new material for the in situ remediation process. In their study, bentonite has been proven to be an effective dispersant and stabilizer in the encapsulation process of nZVI nanoparticles, which reduced aggregation and increased the efficiency of nZVI in the removal of Cr(VI). As the nZVI nanoparticles are stabilized, they remain stable in suspensions for a prolonged period, which facilitates its transport into soil and enhances remediation processes [26].

The surface coating of nanoparticles remains a major challenge due to their high surface energy and the increased surface area of the nanoparticles [27]. In addition, the physiochemical conditions of the soil also play an important role in the remediation processes of heavy metal ions. Even with the application of stabilized nZVI, factors like pH, ionic strength of the electrolyte solution, residence time, and pore water velocity will greatly affect the surface charge and stability of nZVI nanoparticles as they transport through the soil. Most heavy metals contamination studies have focused on batch sorption experiments under equilibrium conditions. However, batch experiments do not provide a full understanding of the factors controlling the transport of heavy metals in soil. Therefore, the transport of heavy metals should also be investigated using a column transport system under variable flow velocities, which better represents the transport of heavy metals under field conditions. Column transport experiments also allow the application of numerical modeling to simulate measured breakthrough curves (BTC) with both linear and non-linear sorption models under equilibrium and non-equilibrium conditions.

Therefore, the goal of this research was to assess the efficiency of the application of CMC-stabilized nZVI nanoparticles for the remediation of Cr(VI) from contaminated soil through sets of batch and column transport experiments under both equilibrium and non-equilibrium conditions. The HYDRUS-1D model was used to investigate the sorption and transport of Cr(VI) under variable saturation conditions and to correlate the experimental data to simulation results. The specific research objectives were to (1) evaluate the effect of pH and IS of CMC-stabilized nZVI nanoparticle suspensions on the reduction and removal efficiency of Cr(VI) from a contaminated loamy sand soil; (2) evaluate the effect of CMC-stabilized nZVI nanoparticles on the transport of Cr in soil columns under variable pore water velocities; and (3) simulate sorption and transport of Cr(VI) through the soil under equilibrium and physical (dual-permeability) and chemical (two-site sorption) non-equilibrium conditions.

## 2. Materials and Methods

### 2.1. Soil Sampling and Analysis

The soil sample was collected from the top 30 cm of the soil surrounding the industrial area (24°32′58.1″ N and 46°55′30.0″ E) in the south of Riyadh city. Previous studies have indicated that these soils are highly contaminated with several heavy metal contaminants including Cr [14]. The soil sample was air-dried and the fine soil was separated using a 2-mm sieve. Standard procedures were carried out to determine the physical and chemical properties of the collected soil sample [28]. The particle size distribution was determined by the pipette method [29]. The soil moisture characteristics were determined by the pressure plate apparatus in the water potential range of 10–1500 kPa [30]. The saturated hydraulic conductivity was determined by the constant-head method [31]. The mineral composition of the soil samples was examined with an X-ray diffractometer, (Shimadzu XRD 7000, Kyoto, Japan). A surface area and microporosity analyzer (ASAP 2020, Micromeritics, Norcross, GA, USA) was used to measure the specific surface area of the soil using the Brunauer, Emmett, and Teller (BET) method and nitrogen (N_2_) at 77 K as the sorbate gas. The zeta potential of the collected soil sample was measured using Zetasizer Nano ZS Series Instrument (Malvern Instrument Ltd., Worcestershire, UK). The soil was sieved through a 150 µm filter and a 250 mg L^−1^ soil suspension was prepared using DI water. The soil suspension was left for 48 h to settle down and the supernatant was collected. Twenty zeta potential readings of the soil suspension were measured, and the average value was recorded. The concentration of Cr(VI) in the collected soil sample was determined by the 1,5-diphenylcarbazide colorimetric method (USEPA method 7196A). The absorbance was measured at a wavelength of 540 nm using a UV/VIS Spectrophotometer (Genesys 10S UV-VIS, Thermo Scientific, Waltham, MA, USA). The total Cr concentration was measured using ICP-AES (Perkin Elmer, Shelton, CT, USA) after acid digestion of soil samples [32].

The physical and chemical properties of the collected soil sample are presented in Table 1. Analysis of Cr(VI) in the collected soil showed that the concentration of Cr(VI) was 7.4 mg∙kg^−1^. This concentration, despite still beg toxic to plants and humans, was deemed low for accurate assessment of the efficiency of CMC-nZVI in the reduction in Cr(VI) in the soil. Therefore, a decision was made to spike the collected soil with a larger concentration of Cr(VI). Four portions of 500 g from the soil were spiked by mixing 500 mL of K_2_Cr_2_O_7_ solution with a concentration of 100 mg L^−1^ [1]. The mixture was sealed in a plastic container and left for static aging for one week in the laboratory (23 ± 2.0 °C). After one week, the mixture was dried in the oven at 40 °C, ground to pass through a 2-mm sieve, and the four portions were thoroughly mixed to form one soil sample. The homogeneity of the spiked soil and the final Cr(VI) concentration were tested using several samples. The final Cr(VI) concentration was found to be 99 ± 0.85 mg∙kg^−1^.

### 2.2. Synthesis and Characterization of Stabilized nZVI Nanoparticles

Zero-valent iron nanoparticles (nZVI) were synthesized by the chemical reduction method using sodium borohydride (NaBH_4_) as the reducing agent [25], according to the following reaction:

(1) $$Fe{\left(H_{2}O\right)}_{6}^{3+}+3BH_{4}^{-}+3H_{2}O\to Fe^{\circ }↓+3B{\left(OH\right)}_{3}+10.5H_{2}$$

The stabilization of nZVI nanoparticles with the carboxymethylcellulose (CMC) polymer was carried out during the preparation. All chemicals used were of analytical grade, with high purity (>99.9). All chemicals were purchased from local suppliers. The CMC-nZVI nanoparticles were synthesized in a 500 mL flask reactor with three open necks. An aqueous solution of 0.1 M iron chloride (FeCl_3_∙6H_2_O, dissolved in a mixture of 4:1 (*v*/*v*) of absolute ethanol (99.9%) to DI water) was added to a 5 g L^−1^ of CMC solution and a mechanical stirrer was housed in one neck of the reactor to ensure vigorous stirring (speed of 150 rpm) of the solution for 30 min. A solution of 0.2 M sodium borohydride (NaBH_4_, dissolved in DI water) was introduced to the mixture dropwise through the second neck of the reactor. The third neck was used to purge N_2_ gas throughout the preparation time. Stirring of the mixture continued (speed of 150 rpm) for another 30 min to ensure the completion of the reaction. The nZVI nanoparticles were collected by vacuum filtration, washed three times with ethanol (99.9%), rinsed with deoxygenated DI water, and dried in an oxygen-free chamber.

Dynamic light scattering (DLS) techniques were used to measure the hydrodynamic size of the prepared CMC-nZVI nanoparticles. Sodium bicarbonate (NaHCO_3_) was used as the background electrolyte solution in all experiments. Two concentrations were used for the NaHCO_3_ background electrolyte solution (0.01 and 0.001 M). The zeta potential of the prepared CMC-nZVI nanoparticle suspensions was quantified by measuring the electrophoretic mobility on a Zetasizer Nano ZS Series Instrument (Malvern Instrument Ltd., UK). The concentration of the CMC-nZVI nanoparticles was determined using a UV/VIS spectrophotometer at 508 nm wavelength (Genesys 10S UV-VIS, Thermo Scientific) [33]. A calibration curve was developed for every measurement by diluting the freshly prepared CMC-nZVI nanoparticle suspension to known aqueous concentrations. The synthesized CMC-nZVI nanoparticles were dried in an oven at 60 °C and the dry powder was used to determine the specific surface area of the CMC-nZVI nanoparticles by the BET method (ASAP 2020, Micromeritics, Norcross, GA, USA). A Transmission Electron Microscope (TEM–JEM1011, JEOL, Inc., Peabody, MA, USA) was used to characterize the structure and morphologies of the synthesized CMC-nZVI nanoparticles.

The total interaction energy on the surfaces of the synthesized CMC-nZVI nanoparticles were calculated using the classical Derjaguin, Landau, Verwey, and Overbeek (DLVO) theory. The DLVO calculates the total interaction energy of the particles, calculated as the sum of the van der Waals attractive and the electric double layer repulsive forces that exist between CMC-nZVI nanoparticles. The interaction between two CMC-nZVI nanoparticles was considered to be a sphere–sphere interaction. The details of the calculations of ionic strength of the background electrolyte solution, the Debye–Huckel length, and the equations used for the calculation of the DLVO interactions are presented in Appendix A.

### 2.3. Batch Experiments of Cr(VI) Reduction in the Soil

The reduction/precipitation mechanism for the removal of Cr(VI) by nZVI nanoparticles results in the production of iron and chromium hydroxides according to the following equations [34]:

(2) $$CrO_{4}^{2-}+Fe^{o}+8H^{+}\;\to \;Fe^{3+}+Cr^{3+}+4H_{2}O$$

(3) $$\left(1-x\right)Fe^{3+}+xCr^{3+}+2H_{2}O\;\to \;Fe_{\left(1-x\right)}Cr_{x}OOH↓+3H^{+}$$

Batch experiments were carried out at 25 ± 2 °C in the laboratory to evaluate the efficacy of stabilized CMC-nZVI nanoparticles for the reduction in Cr(VI) in the collected soil. In total, 2 g of the soil (initial Cr(VI) was 100 mg∙kg^−1^, see Section 3.1 in Results and Discussion) were transferred to polyethylene 50 mL Falcon tubes and 20 mL (ratio of 1:10) of CMC-nZVI suspensions was added. Eight concentrations (0, 10, 50, 100, 150, 250, 350, and 450 mg L^−1^) of the CMC-nZVI nanoparticles were used to determine the concentration at which the maximum reduction in Cr(VI) occurs. CMC-nZVI nanoparticle suspensions were prepared in an electrolyte solution of 0.01 and 0.001 M NaHCO_3_. The pH of the prepared suspensions was adjusted to 5 and 7 with 0.1 M of HCl or NaOH solutions. The designated pH was monitored throughout the experiment and was checked again at the end of the shake time before measurement. The reaction mixture was allowed to react for 2 h with continuous shaking at 150 rpm. After that, the samples were centrifuged at 5000 rpm for 15 min and the supernatant was collected and filtered using 0.45 µm PTFE syringe filter.

The residual Cr(VI) in the filtrate and the total Cr were determined according to the procedure mentioned before. The removal efficiency of Cr(VI) was calculated according to the following equation [35]:

(4) $$Cr\;removal\;efficiency\;\left(\%\right)=\left(1-\frac{C_{e}}{C_{i}}\right)\times 100$$

where *C_i_* and *C_e_* are the initial and supernatant concentrations of Cr(VI), respectively. The removal capacity of the CMC-nZVI nanoparticles was calculated as

(5) $$Removal\;capacity\;\left(Q_{e}\right)=\frac{V\left(C_{i}-C_{e}\right)}{m}$$

where *V* is the volume of the aqueous solution (L) and *m* is the mass of adsorbent (g).

To assess the reaction kinetics of Cr(VI) removal, batch experiments were repeated at the above-mentioned conditions (CMC-nZVI concentration of 250 mg L^−1^) and the analysis was carried out at 15, 30, 60, 90, 120, and 180-min time intervals. Three kinetic models were used to describe the reduction in Cr(VI) by CMC-nZVI nanoparticles: the pseudo-first-order, pseudo-second-order, and the Langmuir–Hinshelwood first-order kinetic models. The three kinetic models were mathematically presented according to the following equations [36,37]:

(6) $$ln\left(q_{e}-q_{t}\right)=ln\left(q_{e}\right)-K_{1}t$$

(7) $$\frac{t}{q_{t}}=\frac{1}{2}K_{2}{q_{e}}^{2}+\frac{t}{q_{e}}$$

(8) $$ln\left(C_{e}/C_{i}\right)=-K_{3}t$$

where *q_e_* and *q_t_* (mg g^−1^) are the removal capacity at equilibrium and at time *t* (min), *K*_1_ (min^−1^), *K*_2_ (g mg^−1^ min^−1^), and *K*_3_ (min^−1^) are the rate constants of the pseudo-first-order, the pseudo-second-order, and the Langmuir–Hinshelwood first-order adsorption, respectively, and *t* is the remediation time (min).

### 2.4. Column Experiments

The effect of the CMC-nZVI nanoparticles on the removal of Cr(VI) was also investigated using an acrylic soil column with 2.5 cm I.D. and 15 cm length. The column was wet-packed (2% *w*/*w*) with 110 g of the collected soil (bulk density of 1.494 g cm^−3^). The column was packed in 3-cm increments to eliminate particle segregation. The column was sealed from the bottom and a nylon membrane (0.45 μm pore opening) was used to prevent passage of soil particles. The hydrodynamic dispersion coefficient was estimated from breakthrough curves obtained with an inert tracer (Cl^−^). A peristaltic pump was used to pre-condition the soil column by flushing 10 PV of DI water from the bottom at a constant flow of 2.0 cm h^−1^. By the end of the pre-condition time, a leachate volume was collected and the concentration of Cr(VI) was determined to assess any change in the initial concentration of Cr(VI) in soil.

The transport experiment started by pumping 10 PV of Cr(VI) solution (100 mg L^−1^) as a pulse input into the system from the bottom. The Cr(VI) solution was pumped at two flow rates (2 and 4 cm h^−1^). This was followed by the application of 10 PV of the CMC-nZVI nanoparticle suspension (250 mg L^−1^, prepared in NaHCO_3_ electrolyte solution at 0.001 and 0.01 M). The prepared suspensions were adjusted to pH 5 and 7 using 0.1 M of HCl or NaOH solutions. Two flow rates (2.0 and 4.0 cm h^−1^) were used to pump 10 PV of the CMC-nZVI suspensions through the column. The CMC-nZVI suspensions were sonicated for 10 min before use. Finally, the column was flushed with DI water until the final time for the simulation (60 h). A fraction collector was used to collect the outflow from the column. The leachate collected was filtered using a 0.45 µm PTFE syringe filter and analyzed for Cr(VI) and total Cr according to the methodology mentioned before. All experiments were carried out in the laboratory (23 ± 2 °C) and each experiment was repeated three times. Concentration data were presented as relative concentration and breakthrough curves (BTCs) were plotted as a function of time.

### 2.5. Simulation of Cr Removal and Transport

Transport parameters for the collected soil sample were determined by fitting the experimental data of the transport of a conservative tracer (Cl^−^) using the CXTFIT model [38]. The CXTFIT model provides an analytical solution for one-dimensional transport based on the convection–dispersion equation (CDE), which is written at equilibrium as:

(9) $$\frac{\partial c}{\partial t}+\rho \frac{\partial s}{\partial t}=\frac{\partial }{\partial z}\left(\theta D\frac{\partial c}{\partial z}\right)-\frac{\partial qc}{\partial z}\pm \emptyset$$

where *C* (mg L^−1^) is the concentration of solute in liquid phase; *t* (h) is the time; *S* (mg g^−1^) is the amount of solute sorbed; *D* (cm^2^ h^−1^) is the hydrodynamic dispersion coefficient; *Z* (cm) is the soil depth; *q* (cm h^−1^) is the volumetric fluid flux density; *ρ* (g cm^−3^) is the soil bulk density; *ϴ* (cm^3^ cm^−3^) is the volumetric water content; and ∅ is a sink term related to biological or chemical reactions other than sorption. The hydrodynamic dispersion coefficient (*D*) of the soil was estimated by the nonlinear least-squares parameters optimization method using observed BTC values of the conservative tracer. Soil dispersivity (λ) was estimated based on the values of the hydrodynamic dispersion coefficient (λ=Dv).

The transport of Cr(VI) in soil columns was simulated using the HYDRUS-1D model [39]. The HYDRUS-1D model can be applied to both equilibrium and non-equilibrium transport in soil. Assuming no degradation of the solute, the physical and chemical non-equilibrium model (also known as the dual-permeability model with two-site sorption) can be presented as

(10) $$\frac{\partial C}{\partial t}+\left(\frac{\rho }{\theta }\right)\left(\frac{\partial S_{1}}{\partial t}+\frac{\partial S_{2}}{\partial t}\right)=D\frac{\partial^{2}C}{\partial Z^{2}}-V\frac{\partial C}{\partial Z}$$

where *S_1_* is the solid phase concentration on equilibrium sites (mg g^−1^). *S*_2_ is the solid phase concentration on kinetic non-equilibrium sites (mg g^−1^).

### 2.6. Statistical Analysis and Quality Control

All experiments were conducted in three replicates. Statistical analysis was carried out using the SPSS software (version 21, SPSS Inc., Chicago, IL, USA). Data values were presented as averages along with its standard deviation (±1SD). The least significant difference (LSD at *p* < 0.01) test was applied to compare the significant differences among the means of the three replicates.

## 3. Results and Discussions

### 3.1. Chemical and Physical Properties of the Collected Soil

The specific surface area of the collected soil sample as determined by the BET method was 3.652 ± 0.004 m^2^ g^−1^. The t-Plot micropore area was very limited and reached 0.114 m^2^ g^−1^, the Barrett–Joyner–Halenda (BJH) adsorption/desorption cumulative volume of pores was 0.0126 cm^3^ g^−1^ and the average pore diameter was 138.0 nm. Adsorption/desorption isotherms showed that the maximum quantity adsorbed of the N_2_ gas was 8.143 cm^3^ g^−1^ STP. Adsorption/desorption isotherms of the collected soil were of type II adsorption isotherms. The soil moisture characteristics curve was obtained by fitting of measured retention data to the van Genuchten equation using the RETC model [40]. Results showed that the water content at saturation, field capacity, and wilting point were 0.38, 0.11, and 0.017 cm^3^ cm^−3^, respectively. The hydraulic parameters of the van Genuchten equation for the collected loamy sand soil were *ϴ_s_* = 0.3817 cm^3^ cm^−3^, *ϴ_r_* = 0.0447 cm^3^ cm^−3^, *α* = 0.0377 cm^−1^, and *n* = 1.73.

### 3.2. Characterization of the Synthesized nZVI Nanoparticles

#### 3.2.1. BET Surface Area and Microporosity

**Figure 1:**
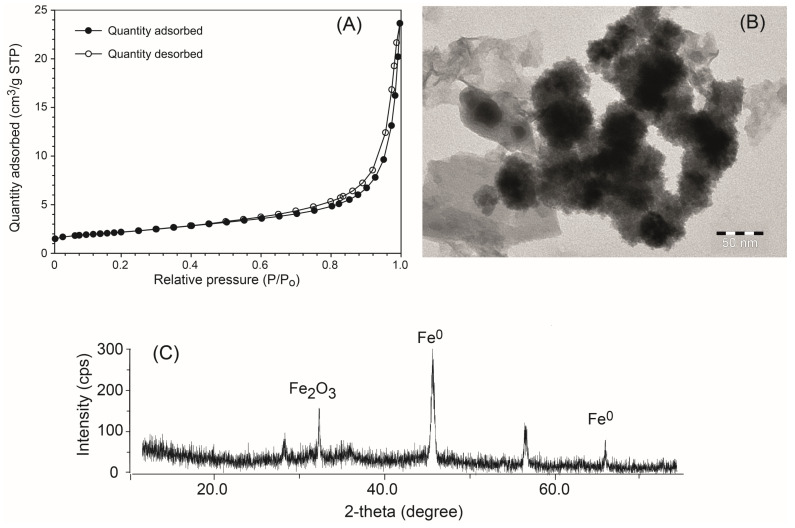
Adsorption/desorption isotherms (type II adsorption isotherms), (**A**); Transmission Electron Microscope (TEM), (**B**); and XRD, (**C**), of the synthesized CMC-nZVI nanoparticles.

The specific surface area of the synthesized CMC-nZVI nanoparticles was 7.805 ± 0.007 m^2^ g^−1^. Previous research has reported specific surface areas of stabilized CMC-nZVI nanoparticles in the range 6.7–12.2 m^2^ g^−1^ [21]. The micropore area represented about 19.6% (1.531 m^2^ g^−1^) out of the total specific surface area of the CMC-nZVI nanoparticles. The BJH adsorption/desorption cumulative volume of pores of the CMC-nZVI nanoparticles was 0.0157 cm^3^ g^−1^ and the average pore diameter was 80.4 nm. The maximum quantity adsorbed of the N_2_ gas based on the adsorption/desorption isotherms was 23.821 cm^3^ g^−1^ STP (Figure 1A). CMC-nZVI adsorption/desorption isotherms were type II adsorption isotherms and demonstrated a very narrow hysteresis loop, which was fully closed at a relative pressure of 0.48. The structure and morphologies of the CMC-nZVI nanoparticles, as determined by the TEM, are presented in Figure 1B. TEM analysis showed that the average size of bare and CMC–nZVI nanoparticles ranged between 50 and 70 nm. XRD analysis showed the presence of Fe^0^ as the dominant form in the synthesized CMC-nZVI nanoparticles (Figure 1C), as confirmed by the characteristic diffraction peaks at 2θ = 45.46° and 65.64°. Some iron oxide was also present at 2θ = 32.6°.

#### 3.2.2. Hydrodynamic Size and Zeta Potential

The measurement of the hydrodynamic size of CMC-nZVI nanoparticles using DLS determines the equivalent size diameter of any particle (or aggregate of particles) within a certain size. Therefore, chemical conditions (e.g., pH and IS) in the CMC-nZVI suspension will greatly affect the hydrodynamic size of the synthesized nanoparticles. In this research, we used two pH (5 and 7) and two IS (0.01 and 0.001 M) values. Therefore, we had four treatments of CMC-nZVI nanoparticle suspensions: pH 5 and 0.01 M; pH 5 and 0.001 M; pH 7 and 0.01 M; and pH 7 and 0.001 M, referred to hereafter as CMC-nZVI 1; CMC-nZVI 2; CMC-nZVI 3; and CMC-nZVI 4, respectively. The average hydrodynamic size of CMC-nZVI nanoparticles at pH 5 and 7 were 130.5 and 73.7 nm, respectively (Table 2). The decrease in IS of the background electrolyte solution from 0.01 to 0.001 M resulted in a slight decrease in the size of the CMC-nZVI nanoparticles by 1.1 and 6.7% at pH 5 and 7, respectively. In contrast, a much larger impact of pH on the hydrodynamic size of CMC-nZVI nanoparticles was observed. The increase in pH of the CMC-nZVI suspension from 5 to 7 resulted in a reduction in the hydrodynamic size by 42.0 and 45.1% at IS values of 0.01 and 0.001 M, respectively. The reduction in the hydrodynamic size of the CMC-nZVI nanoparticles at pH 7 can be related to the larger zeta potential observed at pH 7, which resulted in greater particle stability and minimum particle aggregation.

**Figure 2:**
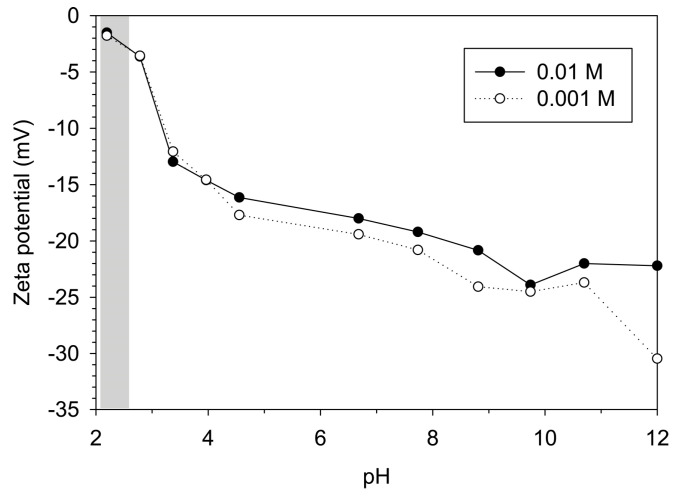
Effect of the IS (0.01 and 0.01 M) of the background electrolyte solution (NaHCO_3_) on the zeta potential of CMC-nZVI nanoparticles (CMC-nZVI concentration of 250 mg L^−1^). The shaded area represents the pH range of the point of zero electric charge (PZC).

The point of zero electric charge (PZC) was measured by measuring the zeta potential of CMC-nZVI in a series of suspensions having IS of 0.01 and 0.001 M and pH range from 2 to 12 (the background electrolyte solution was NaHCO_3_). Regardless of the IS values, the PZC of the synthesized CMC-nZVI nanoparticles was about pH 2 (Figure 2). The average zeta potential of the CMC-nZVI was increased by 9.7% as the pH of the suspension was increased from 5 to 7. On the other hand, under our experimental conditions (i.e., the pH range 5–7), reducing IS from 0.01 M to 0.001 M increased the zeta potential of the CMC-nZVI nanoparticles by 10.1% (Table 2). Decreasing the IS of the background electrolyte solution increases the zeta potential of the CMC-nZVI nanoparticles due to the expansion in the extent of the electrical double layer at lower IS and the subsequent increase in the electrostatic repulsion forces [41].

#### 3.2.3. Total Interaction Energy (DLVO)

**Figure 3:**
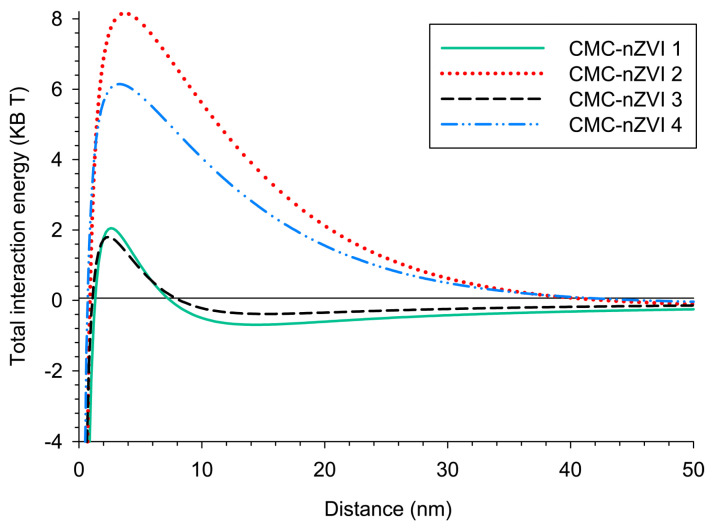
Effect of pH and IS on the total interaction energy of CMC-nZVI suspensions. The background electrolyte solution is NaHCO_3_ and the CMC-nZVI concentration is 250 mg L^−1^.

The classical DLVO theory calculates the total interaction energy between CMC-nZVI nanoparticles; hence, it can be used to assess the stability of the particles based on existing solution chemistry conditions. At the lower IS (0.001 M), larger energy barriers (6.15 to 8.16 K_B_T) were formed between CMC-nZVI nanoparticles indicating unfavorable attachment environments (i.e., domination of electrostatic repulsion forces). The energy barriers diminished to a much smaller range (1.81 to 2.05 K_B_T) with the increase in IS to 0.01 M (Figure 3). Examining the total interaction energy of the different CMC-nZVI suspensions revealed that only the suspensions CMC-nZVI 1 and CMC-nZVI 3 showed the formation of a small secondary minimum at separation distances of 14 and 12 nm, respectively. These are the two CMC-nZVI suspensions with the larger 0.01 M IS. This can be explained by the fact that the electric double-layer interaction is strongly dependent on the IS of the solution. As the IS of the electrolyte solution increases, the energy barrier decreases, and reversible aggregation starts in the secondary minimum. At separation distances of 3 nm (CMC-nZVI 1 and CMC-nZVI 3) and 5 nm (CMC-nZVI 2 and CMC-nZVI 4), the energy barrier is eliminated, and particles irreversibly aggregate in the primary energy minimum as a result to the domination of the van der Waals attraction forces (Figure 3).

In general, under our experimental conditions, the energy barrier was decreased as the pH was increased from 5 to 7 and the IS was increased from 0.001 to 0.01 M. The ranking order of the decrease (i.e., less particle stability) in the energy barriers was CMC-nZVI 2 > CMC-nZVI 4 > CMC-nZVI 1 > CMC-nZVI 3. These findings indicate that the stability of the CMC-nZVI nanoparticles is controlled by pH followed by the IS of the suspension. According to the DLVO theory, energy barriers around 20 K_B_T are required for particles to remain stable in suspension for a long time. The largest energy barrier that was observed with the suspension CMC-nZVI 2 reached only 8.16 K_B_T. Therefore, this energy barrier can easily vanish with the decrease in the separation distance between the particles and the subsequent increase in the extent of the van der Waals attractive forces.

### 3.3. Effect of the Concentration of CMC-nZVI on the Reduction Efficiency of Cr(VI)

**Figure 4:**
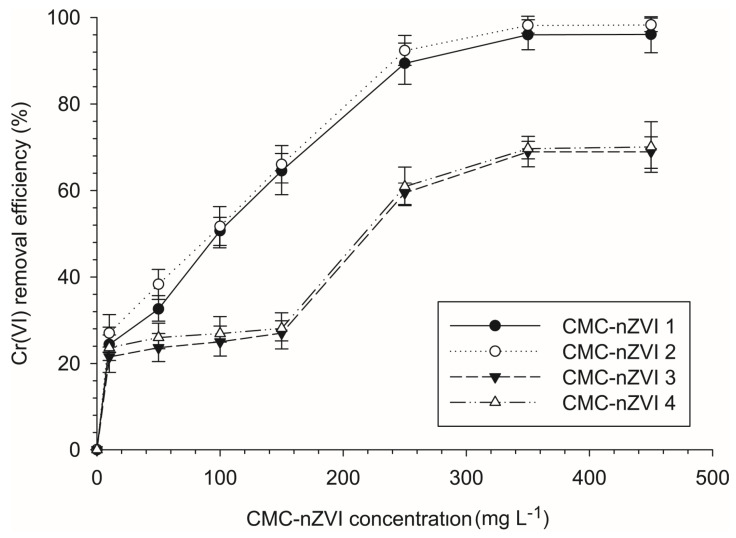
Effect of the concentration of CMC-nZVI nanoparticles on the removal efficiency of Cr(VI). The vertical bars represent the standard deviation of the measurements.

The removal efficiency of Cr(VI) after 2 h of contact time ranged between 21.5 and 98.3% (Figure 4). Increasing the concentration of the CMC-nZVI nanoparticles from 10 to 250 mg Kg^−1^ greatly enhanced the removal efficiency. The average removal efficiency of Cr(VI) was increased from 24.1 to 75.5% when the concentration of CMC-nZVI nanoparticles was increased from 10 to 250 mg L^−1^, respectively. The increase in the concentration of CMC-nZVI provides a larger number of particles, hence more surfaces and a larger sum of total surface area for the reaction of Cr(VI) reduction can take place, which improves the removal efficiency. A similar conclusion was also mentioned by Gueye et al. [42] who reported that the increase in the Cr removal with increasing the concentration of Fe^0^ was attributed to the increase in the reactive sites associated with larger Fe^0^ concentrations.

Further increases in the concentration of CMC-nZVI nanoparticles to 350 and 450 mg L^−1^ resulted in only a slight increase (83.2 and 83.4%, respectively) in the average removal efficiency of Cr(VI). At larger CMC-nZVI nanoparticle concentrations, the stability of the particles is reduced and particle agglomeration starts to form larger-size aggregates. The increase in the hydrodynamic size of CMC-nZVI nanoparticles due to the formation of larger-sized aggregates will reduce the total surface area of the particles. Therefore, it is not anticipated that the sum of the total surface area of CMC-nZVI nanoparticles will significantly continue to increase as the concentration of CMC-nZVI nanoparticles increases above a certain concentration. Figure 4 shows that the removal efficiency of Cr(VI) almost leveled at a CMC-nZVI concentration of 250 mg L^−1^ (at all our experimental pH and IS values). Therefore, the CMC-nZVI concentration of 250 mg L^−1^ was considered the optimum concentration for the removal of Cr(VI) from the loamy sand-contaminated soil used in this study.

### 3.4. Effect of Contact Time on the Reduction Efficiency of Cr(VI)

**Figure 5:**
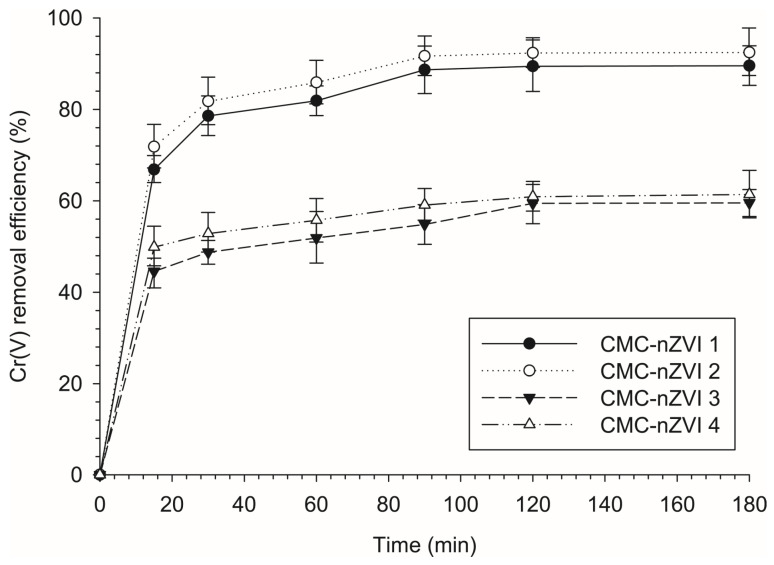
Effect of contact time of CMC-nZVI nanoparticles on the removal efficiency of Cr(VI). The vertical bars represent the standard deviation of the measurements.

The reaction rate of Cr(VI) removal by CMC-nZVI nanoparticles (250 mg L^−1^) increased with the increase in contact time. The removal efficiency of Cr(VI) reached 48.8–81.8% after 30 min (Figure 5). The average removal efficiency of Cr(VI) reached 68.9, 73.6, 75.5, and 75.8% at contact times of 60, 90, 120, and 180 min, respectively. These results indicate that the reaction rate was fast within the first 30 min and gradually slowed down until it was almost leveling after 2 h. The maximum removal efficiency of Cr(VI) reached 59.5–92.4% after 2 h. of contact time. The average removal efficiency of Cr(VI) reached 90.1 and 60.5% at pH 5 and 7, respectively, indicating favorable reduction for the Cr(VI) at lower pH values. Increased release of Fe^2+^ under acidic conditions suppresses the formation of iron oxide on the surfaces of the CMC-nZVI nanoparticles, hence, enhancing the reduction in Cr(VI). Our results are consistent with Du et al. [12] who reported that using 6% (*w*/*w*) nZVI nanoparticles in chromium ore processing residue containing 15.9 mg∙kg^−1^ Cr(VI) resulted in a complete reduction in Cr(VI). In contrast, Singh et al. [20] reported that it took 40 days to reach 99% of remediation efficiency of Cr(VI) in soil contaminated by tannery wastes (Cr(VI) = 43.3 mg∙kg^−1^) when 5 g L^−1^ nZVI suspension was used for the in situ remediation.

### 3.5. Effect of pH and Is on the Reduction Efficiency of Cr(VI)

**Figure 6:**
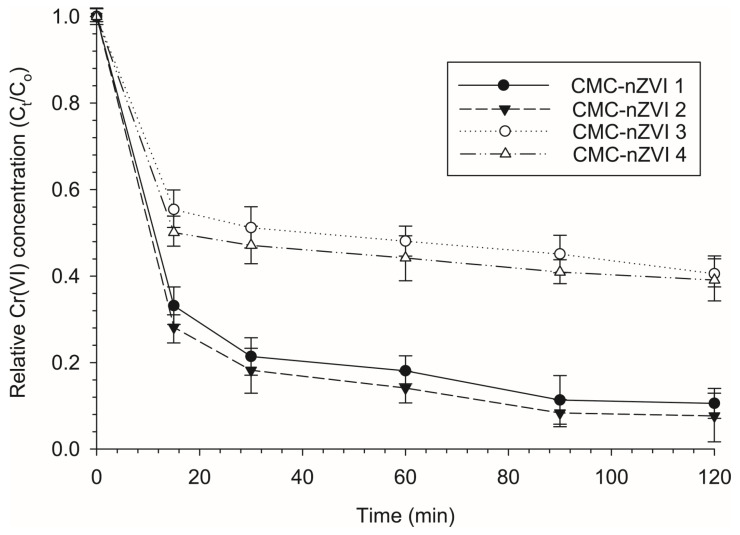
Effect of pH and IS of the CMC-nZVI suspensions on the relative concentration of Cr(IV) in the contaminated loamy sand soil. The vertical bars represent the standard deviation of the measurements.

The removal efficiency of Cr(VI) was greatly affected by the initial pH of the CMC-nZVI suspension. The increase in pH from 5 to 7 resulted in a large decrease in the efficiency of Cr(VI) removal. After two hours of contact time, reducing the pH of the CMC-nZVI suspension from 7 to 5 decreased the average relative concentration of Cr(VI) by 73.9 and 80.4% at IS of 0.01 and 0.001 M, respectively (Figure 6). The decrease in the relative concentration of Cr(VI) indicates an enhancement in the removal efficiency of Cr(V) from the contaminated soil. These results confirm the negative correlation between the removal efficiency of Cr(VI) and pH value [43,44,45]. In general, the removal efficiency of Cr(VI) from contaminated soil is much larger under acidic conditions. Due to the release of Fe^2+^ under acidic conditions, the formation of iron oxide on the surfaces of the nZVI nanoparticles is suppressed well, leaving the surfaces of the nZVI particles more active, which accelerates the reduction in Cr(VI) [46].

The effect of IS of the background electrolyte solution on the removal efficiency of Cr(VI) was much less pronounced. After two hours of contact time, reducing the IS of the CMC-nZVI suspension from 0.01 to 0.001 M decreased the average relative concentration of Cr(VI) only by 27.6 and 3.6% at pH values of 5 and 7, respectively (Figure 6). The enhancement in the removal efficiency of Cr(VI) at low IS was attributed to the increase in the charges on the surfaces of the CMC-nZVI nanoparticles. At low IS, the zeta potential of the background electrolyte solution increases (Table 2) due to the expansion of the electrical double layer, which increases the electrostatic repulsion forces [47].

The combined effect of pH and IS on the removal efficiency of Cr(VI) showed that maximum removal efficiency occurred at low pH and IS values. The relative concentration of Cr(VI) at equilibrium in the different CMC-nZVI suspensions reached 0.106, 0.077, 0.405, and 0.391 for CMC-nZVI 1, CMC-nZVI 2, CMC-nZVI 3, and CMC-nZVI 4, respectively. The minimum removal efficiency of Cr(VI) was observed with the CMC-nZVI 3 suspension at pH 7 and IS 0.01 M. Analysis showed that, at pH 7, when the IS of the CMC-nZVI suspension was lowered to 0.001 M, only a slight decrease (3.6%) occurred in the relative concentration of Cr(VI) (i.e., CMC-nZVI 4). Again, this is an indication of the slight impact of changes in the IS on the removal efficiency of Cr(VI). In contrast, lowering the pH to 5 resulted in a decrease in the relative concentration of Cr(VI) by 73.9 and 81.1% at IS values of 0.01 (i.e., CMC-nZVI 1) and 0.001 M (i.e., CMC-nZVI 2), respectively. These findings confirm previous results that showed pH to be the primary controlling factor in the stability and efficiency of CMC-nZVI nanoparticles in the removal of Cr(VI) from contaminated soils.

### 3.6. Adsorption Kinetics of Cr(VI)

The mechanism of the reduction in Cr(VI) was investigated with several kinetic models. The pseudo-second-order was observed as the best-fitted kinetic model for describing the reduction in Cr(VI) on the CMC-nZVI nanoparticles. Larger linear regression coefficients (average *r*^2^ of 0.998) were observed with the pseudo-second-order kinetic model, as compared to the Langmuir–Hinshelwood (average *r*^2^ of 0.956) and the pseudo-first-order (average *r*^2^ of 0.893) kinetic models (Table 3). The fact that *r*^2^ of the pseudo-second-order kinetic model was always larger than 0.99 indicates that the mechanism of Cr(VI) removal was mainly a chemical sorption process [48,49]. In general, larger rate constants were observed at the lower pH (i.e., pH 5) and IS (i.e., 0.001 M) values.

**Figure 7:**
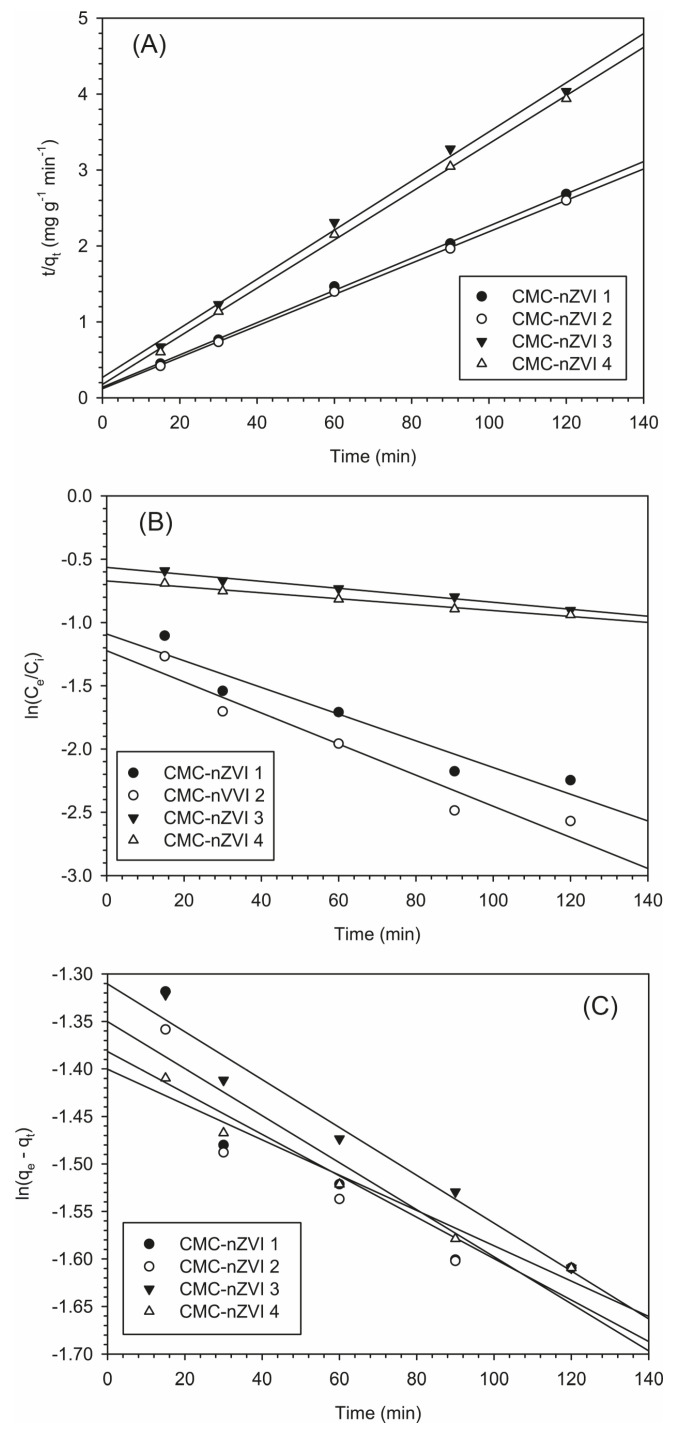
Effect of pH and IS on the reaction kinetics of Cr(VI) using the pseudo-second-order, (**A**); the Langmuir–Hinshelwood first-order, (**B**); and the pseudo-first-order, (**C**) kinetic models. (CMC-nZVI 1, pH 5 and 0.01 M; CMC-nZVI 2, pH 5 and 0.001 M; CMC-nZVI 3, pH 7 and 0.01 M; and CMC-nZVI 4, pH 7 and 0.001 M). Initial Cr(VI) concentration is 100 mg L^−1^, and CMC-nZVI concentration is 250 mg L^−1^.

**Figure 8:**
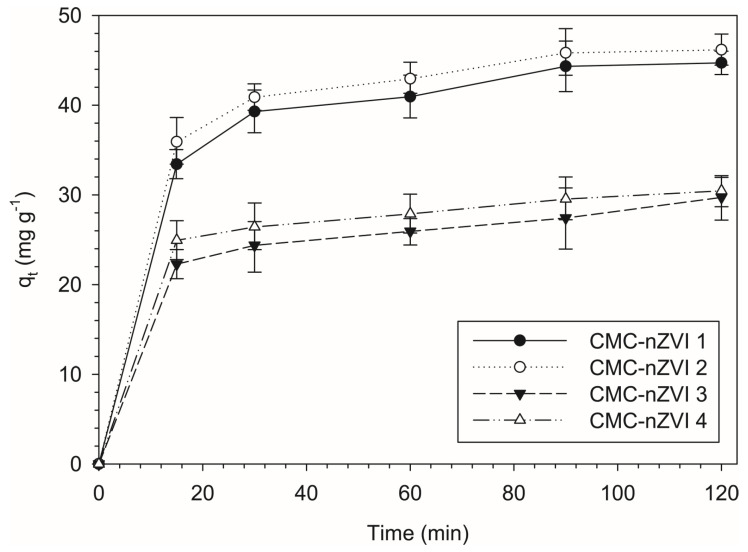
Effect of pH and IS on removal capacity of Cr(VI) (CMC-nZVI 1, pH 5 and 0.01 M; CMC-nZVI 2, pH 5 and 0.001 M; CMC-nZVI 3, pH 7 and 0.01 M; and CMC-nZVI 4, pH 7 and 0.001 M). Initial Cr(VI) concentration is 100 mg L^−1^ and the CMC-nZVI concentration is 250 mg L^−1^. The vertical bars represent the standard deviation of the measurements.

The fittings of the experimental data to the different kinetic models are presented in Figure 7 for the pseudo-second-order, the Langmuir–Hinshelwood first-order, and the pseudo-first-order models, respectively. At approximately 30 min, most of the Cr(VI) was reduced and the surfaces of the CMC-nZVI nanoparticles started to reach dynamic equilibrium with Cr(VI). Based on the pseudo-second-order kinetic model, the plot of *q_t_* (Cr(VI) removal capacity at time *t*) vs time showed a rapid increase in the removal capacity after 30 min from the start of the reaction (Figure 8). This was followed by a gradual leveling of the removal capacity until finally reaching the maximum removal capacity (*q_e_*).

The pH of the CMC-nZVI greatly affected the removal capacity at equilibrium. The average removal capacity at equilibrium was 47.7 and 30.6 mg g^−1^ at pH 5 and 7, respectively, indicating a decrease in the average removal capacity at equilibrium by 35.9% when the pH was increased from 5 to 7. In contrast, no significant changes were observed as a result of changes in IS. Decreasing the IS from 0.01 to 0.001 M only increased the average removal capacity at equilibrium by 2.4% at pH 5. The impact was less profound at pH 7 and the variation in the average removal capacity at equilibrium between the two IS (0.01 and 0.001 M) was only 1.7%. These results are consistent with previous results that showed pH to be the primary factor controlling the reduction in Cr(VI) by CMC-nZVI nanoparticles.

According to the Langmuir–Hinshelwood first-order model, the average half-life for the removal of Cr(V) was 60.9 min at pH 5. This value was increased more than fourfold to an average of 274.5 min at pH 7. The longer half-life for the removal of Cr(VI) from contaminated soil at larger pH values is attributed to the increase in the precipitate of iron hydroxide on the surfaces of the nZVI nanoparticles under alkaline conditions, which decreases the reactivity of the nZVI nanoparticles leading to slower reduction rates [50].

### 3.7. Column Transport of Cr(VI)

The BTC of the nonreactive tracer (CaCl_2_) was fitted in the CXTFIT model using Equation (9). The hydrodynamic dispersion and dispersivity values for the loamy sand soil are presented in Table 4. Increasing the flow rate from 2 to 4 cm h^−1^ greatly increased the hydrodynamic dispersion. The pore water velocity was calculated based on the assumption of steady-state conditions. Larger values for the distribution coefficient (*K_d_*) were observed at pH 5, as compared to *K_d_* values at pH 7. The time required for one pore volume (PV) to pass through the soil column was found to be 2.86 and 1.43 h for the low (2 cm h^−1^) and high (4 cm h^−1^) flow rates, respectively. The parameters of the two-site chemical non-equilibrium model were obtained by fitting Equation (9) to the experimental data using the Hydrus-1D program (Table 5). The good fit obtained (*r*^2^ = 0.96–0.98) at all pH and IS values indicates that the model precisely describes the transport and sorption of Cr(VI) in the loamy sand soil. As the pH of the CMC-nZVI nanoparticle suspension was increased from 5 to 7, a notable decrease in the retardation factor (*R*) was observed. This could be attributed to the increase in the reduction in Cr(VI) to the less mobile and strongly adsorbed Cr(III) at low pH [51].

### 3.8. Modeling the Transport of Cr(VI) (Uniform Model)

**Figure 9:**
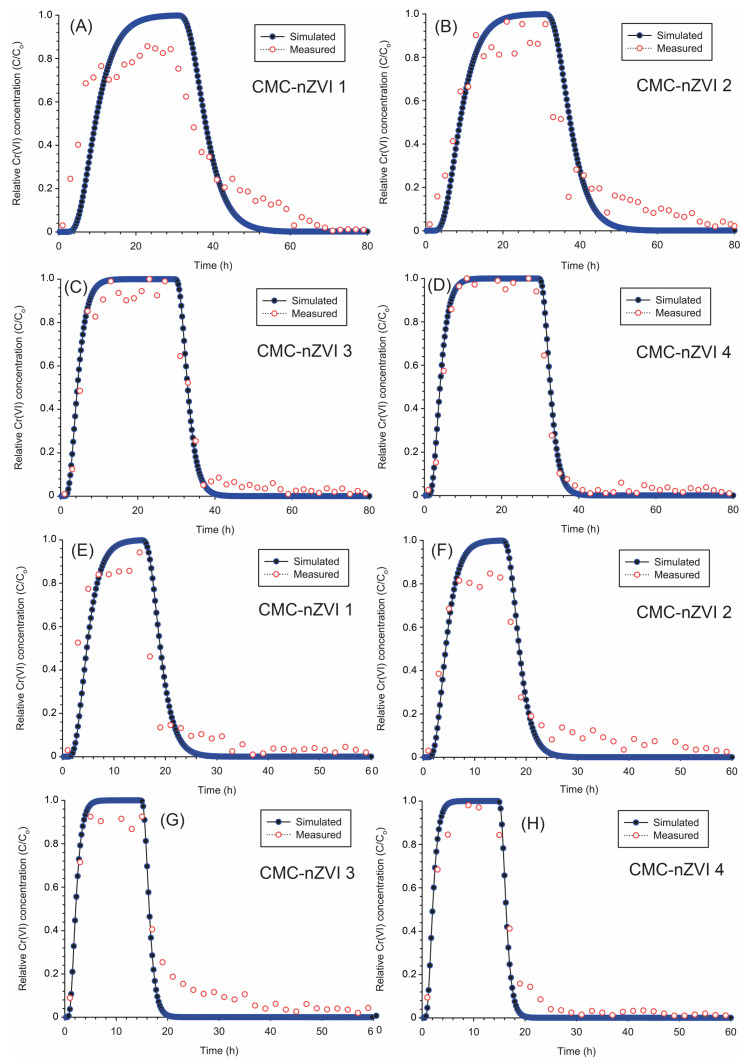
Breakthrough curves for the transport of Cr(VI) (uniform transport model) at a low flow rate of 2 (**A**–**D**) and a high flow rate of 4 (**E**–**H**) cm h^−1^ as they are affected by the pH and IS of the CMC-nZVI suspensions.

Modeling the transport of Cr(VI) under uniform flow conditions revealed that the relative concentration (RC) of Cr(VI) was increased by increasing the pH of the CMC-nZVI suspension from 5 to 7. The average retardation was 3.51 and 1.61 at pH values of 5 and 7, respectively, which could explain the larger transport of Cr(VI) at pH 7. The breakthrough curves (BTC) of Cr(VI) always showed earlier arrival and a shorter time to reach a maximum RC at pH 7 as compared to the same values at pH 5 (Figure 9). For example, simulations based on the flow rate of 2 cm h^−1^ showed that breakthroughs of Cr(VI) occurred after an average of 3.62 and 1.75 h when the pH of the CMC-nZVI suspension was 5 and 7, respectively (Figure 9A–D). Similarly, the average time required to reach maximum RC was 28.75 and 15.75 h when the pH of the CMC-nZVI suspension was 5 and 7, respectively (Table 6).

The observed trend of earlier Cr(VI) arrival and shorter time to reach maximum RC at pH 7 was less pronounced at the high rate of 4 cm h^−1^. BTCs of Cr(VI) occurred after an average of 1.87 and 1.00 h and the average time required to reach maximum RC was 14.75 and 7.75 h at pH 5 and 7, respectively (Figure 9E–H). Under uniform transport, the maximum RC of Cr(VI) was always close to 1.0 (i.e., maximum Cr(VI) concentration in the effluent of 100 mg L^−1^). At pH 7, a maximum RC slightly larger than 1.0 was also observed (Figure 9C,D,G,H). Values of RC larger than 1.0 are related to the underestimation of Cr(VI) sorption and the overestimation of the first-order rate coefficient based on the simulation of the uniform transport model [52].

Variations in pH did not significantly affect the amount of Cr(VI) removed from the soil column. The total amount of Cr(VI) inside the soil column was 39.1 mg (an initial 11 mg was present in the packed soil inside the column and 28.1 mg was added through the 10 PV pulse of the Cr(VI) solution). Out of this amount, an average of 27.4 and 27.5 mg were removed at pH 5 and 7, respectively. When the flow rate was increased from 2 to 4 cm h^−1^, the average amount of Cr(VI) removed was slightly larger and reached 28.3 and 28.4 mg at pH 5 and 7, respectively. Based on the amount removed of Cr(VI) and the simulations of the uniform transport, the removal efficiency of Cr(VI) ranged between 70.3 and 72.6% at the low and high flow rates, respectively (Table 6).

### 3.9. Modeling the Transport of Cr(VI) (Two-Site Sorption Model)

The transport of Cr(VI) in soil is likely to occur under physical and chemical non-equilibrium conditions. Therefore, simulations of the sorption and transport of Cr(VI) in the contaminated loamy sand soil based on the dual-permeability two-site model were more consistent with measured concentrations of Cr(VI) in the leachate collected from the column experiment (Table 7). The overall average for the coefficient of determination (*r*^2^) between measured and simulated Cr(VI) concentrations was 0.81–0.98 and 0.96–0.98 for the uniform and the two-site sorption models, respectively (Table 4 and Table 5). In particular, at pH 5, the two-site model was more accurate (average *r*^2^ = 0.98) in simulating Cr(VI) transport as compared to the uniform transport model (average *r*^2^ = 0.87). This could be explained by the increased sorption of Cr(VI) and the larger retardation that was observed at pH 5, which can lead to less accuracy when the uniform transport model is applied.

**Figure 10:**
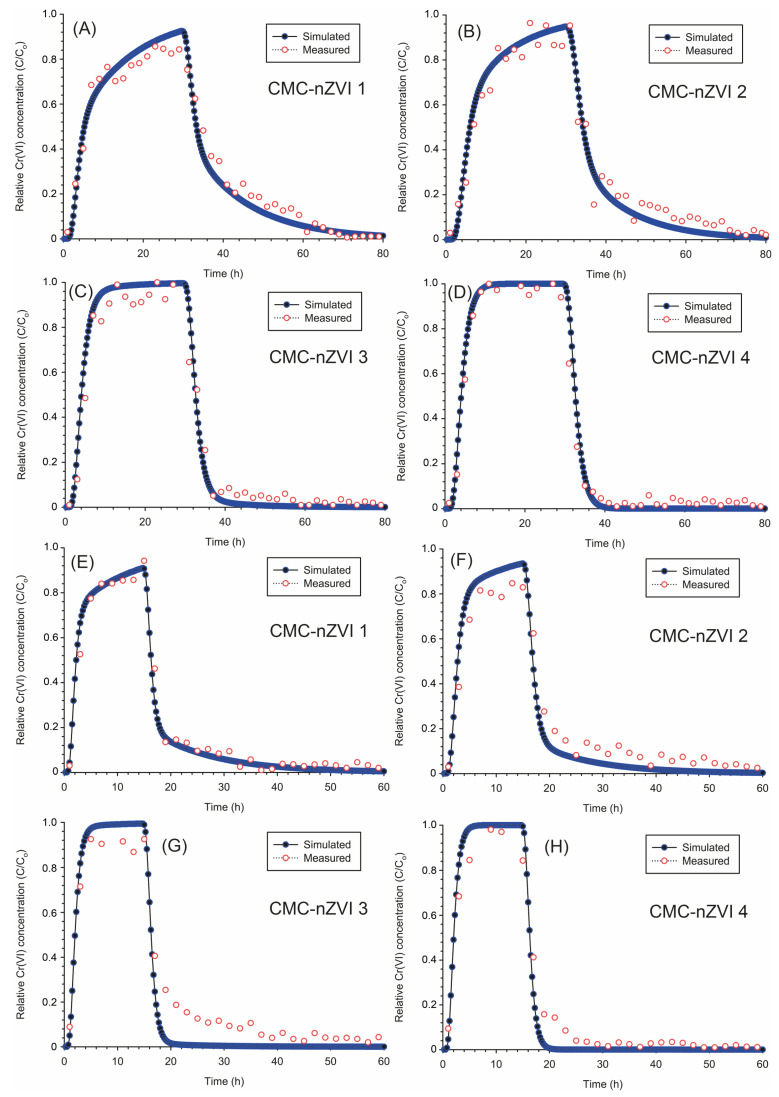
Breakthrough curves for the transport of Cr(VI) (two-site sorption model) at a low flow rate of 2 (**A**–**D**) and high flow rate of 4 (**E**–**H**) cm h^−1^ as they are affected by the pH and IS of the CMC-nZVI suspensions.

Simulations of Cr(VI) transport based on the two-site sorption model showed slightly earlier BTCs and a longer time to reach maximum RC as compared to the simulations of the uniform transport model (Figure 10). For example, at pH 7 and low flow rate, the average time to reach maximum RC was 21.6 and 15.7 h for the two-site sorption (Table 7) and the uniform transport (Table 6) models, respectively. The most significant difference between Cr(VI) simulation based on the two-site sorption and the uniform transport models was observed with the maximum RC. As mentioned earlier, the maximum RC based on the uniform transport model was close to 1.0 at all pH, IS, and flow rate values (Table 6). However, the two-site sorption model showed much less RC and the average RC of Cr(VI) at pH 5 was 0.93 and 0.92 for the flow rates 2 and 4 cm h^−1^, respectively (Table 7). Because of the increased transport of Cr(VI) that was observed at pH 7, RC based on the two-site sorption model at pH 7 remained close to 1.0 at the two flow rates (Figure 10C,D,G,H).

The two-site sorption model takes into consideration both the solid phase concentration on equilibrium sites (*S*_1_) and the solid phase concentration on kinetic non-equilibrium sites (*S*_2_) [53]. Therefore, the shape of the BTC varied between pH 5 and 7. At pH 5, increased sorption and larger retardation resulted in less instantaneous sorption (*S*_1_ sites) and more kinetic sorption (*S*_2_ sites), which led to a slight delay in the Cr(VI) arrival and more pronounced tailing for the BTC (Figure 10A,B,E,F). In contrast, at pH 7, more instantaneous sorption was observed and fast equilibration between the liquid and solid phase concentrations was reached, which led to BTC resembling the uniform transport more (Figure 10C,D,G,H). No significant changes in the amount of Cr(VI) removed and the removal efficiency were observed between the simulation results of the uniform transport and the two-site sorption models. The average amount of Cr(VI) removed was 27.4 and 28.3 mg and the removal efficiency was 70.0 and 72.4% at the low and high flow rates, respectively. Under our experimental conditions, variations in IS of the background electrolyte solution did not significantly affect the transport of Cr(VI) in the contaminated loamy sand soil. In general, a slight increase in the transport of Cr(VI) was observed at the lower IS of 0.001 M but the increase did not exceed 1–3% of Cr(VI) transport at the larger IS of 0.01 M.

### 3.10. Effect of Flow Rate on the Transport of Cr(VI)

The pore water velocity inside the soil column was 5.23 and 10.47 cm h^−1^ at the water flow rates of 2 and 4 cm h^−1^, respectively. The larger flow rate reduced the time required for the arrival of the Cr(VI) almost by half. For example, under uniform transport, the average time required for Cr(VI) arrival was 2.69 and 1.43 h at the water flow rates of 2 and 4 cm h^−1^, respectively (Table 6). Similarly, under non-equilibrium transport, the average time required for Cr(VI) arrival was 1.87 and 1.0 h at the water flow rates of 2 and 4 cm h^−1^, respectively (Table 7). The time required to reach the maximum RC of Cr(VI) was also reduced by almost half as the flow rate was increased from 2 to 4 cm h^−1^.

The maximum RC of Cr(VI) was mainly controlled by the pH of the CMC-nZVI nanoparticle suspension and no significant variations were observed as a result of the increase in the flow rate. For example, at pH 7, the maximum RC of Cr(VI) was always close to 1.0, regardless of the flow rate and/or the transport model (i.e., uniform vs. non-equilibrium). Nevertheless, increasing the flow rate had a limited impact on the amount removed and the removal efficiency of Cr(VI). Increasing the flow rate from 2 to 4 cm h^−1^ increased the removal efficiency by only 3.3 and 3.4% under uniform and non-equilibrium conditions, respectively. The increase in the flow rate will result in larger pore water velocity inside the soil column. Therefore, the tailing of the BTC at pH 5, which is related to more kinetic sorption, was less pronounced (Figure 10C,D).

## 4. Conclusions

Stabilized CMC-nZVI nanoparticles efficiently removed up to 90% of Cr(VI) concentration in contaminated loamy sand soil. Batch experiments showed that the removal efficiency of Cr(VI) from the contaminated loamy sand soil was much larger under acidic conditions. The average removal efficiency of Cr(VI) reached 90.1 and 60.5% at pH 5 and 7, respectively. This is attributed to the increased release of Fe^2+^ under acidic conditions, which suppress the formation of iron oxide on the surfaces of the nZVI nanoparticles and accelerates the reduction in Cr(VI). In contrast, the average removal efficiency of Cr(VI) was slightly larger at lower IS, mainly because of the increased CMC-nZVI nanoparticle stability at lower IS values. These results indicate that pH is the primary controlling factor in the stability and removal efficiency of Cr(VI) by the CMC-nZVI nanoparticles. The experimental results for the reduction in Cr(VI) were best fitted by the pseudo-second-order kinetic model (average *r^2^* of 0.998), indicating that the main mechanism of Cr(VI) removal was by chemical reduction.

Column transport showed that the transport of Cr(VI) was larger at pH 7. The average retardation was 3.51 and 1.61 at pH values of 5 and 7, respectively, which could explain the larger transport of Cr(VI) at pH 7. The breakthrough curves (BTC) of Cr(VI) always showed earlier arrival and a shorter time to reach maximum RC at pH 7 as compared to the same values at pH 5. Simulation of the sorption and transport of Cr(VI) were more accurate when the dual-permeability two-site sorption model was applied. The overall average coefficient of determination (*r^2^*) between measured and simulated Cr(VI) concentrations was 0.81–0.98 and 0.96–0.98 for the uniform and the two-site sorption models, respectively. Simulations of Cr(VI) transport based on the two-site sorption model showed slightly earlier BTCs and longer time to reach maximum RC as compared to the simulations of the uniform transport model. The good fit that was obtained between measured and simulated values at all pH and IS indicates that the Hydrus-1D model adequately describes the sorption and transport of Cr(VI) in the contaminated loamy sand soil. Based on the outcomes of this study, combining column experiment and modeling transport using the Hydrus-1D model can facilitate the assessment of Cr(VI) removal from polluted soils, offering cost efficient and environmentally friendly methodology to remediate soils polluted with heavy metal contaminants.

## Figures and Tables

**Table 1:** Physical and chemical properties of the loamy sand soil used in the study.

Measurement	Unit	Value
Sand	%	81.5
Silt	%	11.4
Clay	%	7.1
Texture	-	Loamy sand
SHC	cm s^−1^	11.4 × 10^−4^
pH	-	7.85
EC	dS m^−1^	1.92
OM	%	0.18
CEC	cmolc∙kg^−1^	6.2
Calcium	meq L^−1^	4.17
Magnesium	meq L^−1^	1.33
Sodium	meq L^−1^	12.54
Potassium	meq L^−1^	1.05
Bicarbonate	meq L^−1^	1.17
Chloride	meq L^−1^	10.60
Sulphate	meq L^−1^	7.32
Cr(VI)	mg∙kg^−1^	7.4

SHC, saturated hydraulic conductivity; pH, potential hydrogen; EC, electrical conductivity; OM, organic matter; CEC, cation exchange capacity.

**Table 2:** Characteristics of the different suspensions of CMC-nZVI nanoparticles.

CMC-nZVI Suspensions	pH	IS (M)	Hydrodynamic Size (nm)	Zeta Potential (mV)
CMC-nZVI 1	5	0.01	131.2 ± 12.5	−16.8 ± 1.2
CMC-nZVI 2	5	0.001	129.8 ± 14.2	−18.1 ± 2.3
CMC-nZVI 3	7	0.01	76.1 ± 9.4	−18.0 ± 1.1
CMC-nZVI 4	7	0.001	71.3 ± 8.5	−20.3 ± 0.9

Carboxymethylcellulose (CMC), zero-valent iron (nZVI), ionic strength (IS). Zeta potential was determined by the measurement of electrophoretic mobility of the synthesized CMC-nZVI nanoparticles and the application of Smoluchowski approximation.

**Table 3:** Kinetic parameters for the removal of Cr(VI) by CMC-nZVI nanoparticles.

Kinetic Model	Parameters	CMC-nZVI Nanoparticles			
		CMC-nZVI 1	CMC-nZVI 2	CMC-nZVI 3	CMC-nZVI 4
Pseudo-first-order	*q_e_*	0.259	0.251	0.269	0.246
	*K*_1_	2.08 × 10^−5^	1.83 × 10^−5^	2.08 × 10^−5^	1.58 × 10^−5^
	*r* ^2^	0.810	0.831	0.967	0.966
Pseudo-second-order	*q_e_*	47.169	48.309	30.864	30.345
	*K*_2_	1.26 × 10^−4^	1.01 × 10^−4^	5.61 × 10^−4^	3.55 × 10^−4^
	*V_o_*	0.282	0.237	0.535	0.354
	*r* ^2^	0.999	0.999	0.994	0.998
Langmuir–Hinshelwood first-order	*K*_3_	10.6 × 10^−3^	12.3 × 10^−3^	2.8 × 10^−3^	2.3 × 10^−3^
	*t*_1/2_	65.3	56.4	247.5	301.4
	*r* ^2^	0.922	0.939	0.981	0.984

CMC-nZVI 1, pH 5 and 0.01 M; CMC-nZVI 2, pH 5 and 0.001 M; CMC-nZVI 3, pH 7 and 0.01 M; and CMC-nZVI 4, pH 7 and 0.001 M. *q_e_*, removal capacity at equilibrium (mg g^−1^); *K*_1_ the rate constant of the pseudo-first-order (min^−1^), *K*_2_ the rate constant of the pseudo-second-order (g mg^−1^ min^−1^) and *K*_3_ is the rate constants of the Langmuir–Hinshelwood first-order model (min^−1^). *V_o_*, the pseudo-second-order initial rate (mg g^−1^ min^−1^) at t = 0 min (calculated as *V_o_ = K*_2_ *q_e_*^2^). *t*_1/2_, half-life time (min) at which half of the initial Cr(VI) is degraded (calculated as *ln*(2)*/K*_3_); *r*^2^, coefficient of determination.

**Table 4:** Transport parameters of the uniform transport were obtained by fitting the equilibrium CDE to the BTCs of a CaCl_2_ tracer and Cr(VI) in CXTFIT and Hydrus models, respectively.

CMC-nZVI Suspension	Water Flow Rate							
	Low (2.0 cm h^−1^)				High (4.0 cm h^−1^)			
	*D*	*λ*	*K_d_*	*r* ^2^	*D*	*λ*	*K_d_*	*r* ^2^
CMC-nZVI 1	8.16	1.56	0.67	0.81	16.33	1.56	0.67	0.85
CMC-nZVI 2	8.11	1.55	0.61	0.88	16.23	1.55	0.60	0.95
CMC-nZVI 3	8.42	1.61	0.17	0.97	16.86	1.61	0.16	0.97
CMC-nZVI 4	8.26	1.58	0.14	0.98	16.54	1.58	0.14	0.97

CMC-nZVI 1, pH 5 and 0.01 M; CMC-nZVI 2, pH 5 and 0.001 M; CMC-nZVI 3, pH 7 and 0.01 M; and CMC-nZVI 4, pH 7 and 0.001 M. CDE, convection–dispersion equation; D, hydrodynamic dispersion (cm^2^ h^−1^) (calculated as *λV*, where *V* is the pore water velocity); *λ*, dispersivity (cm); *K_d_*, the distribution coefficient (ml g^−1^). Pore water velocity was calculated as $\frac{q}{\theta }$ (where *q* is the water flow rate and *ϴ* is the volumetric water content). *r*^2^, coefficient of determination.

**Table 5:** Transport parameters of the physical and chemical non-equilibrium (two-site sorption) obtained by fitting the non-equilibrium CDE to the BTCs of Cr(VI) in the Hydrus-1D model.

CMC-nZVI Suspension	Water Flow Rate							
	Low (2.0 cm h^−1^)				High (4.0 cm h^−1^)			
	*R*	*β*	*ω*	*r* ^2^	*R*	*β*	*ω*	*r* ^2^
CMC-nZVI 1	3.62	0.42	6.02	0.97	3.62	0.42	3.00	0.98
CMC-nZVI 2	3.38	0.58	4.07	0.97	3.34	0.58	2.01	0.96
CMC-nZVI 3	1.67	0.92	0.38	0.97	1.62	0.92	0.19	0.97
CMC-nZVI 4	1.54	0.99	0.04	0.98	1.54	0.99	0.02	0.97

CMC-nZVI 1, pH 5 and 0.01 M; CMC-nZVI 2, pH 5 and 0.001 M; CMC-nZVI 3, pH 7 and 0.01 M; and CMC-nZVI 4, pH 7 and 0.001 M. CDE, convection–dispersion equation; *R*, the retardation factor (calculated as $R=1+\frac{\rho K_{d}}{\theta }$); *β*, the partitioning coefficient (calculated as: $\beta =\frac{\left(\theta +F\rho K_{d}\right)}{\left(\theta +\rho K_{d}\right)}\;$, where *F* is the fraction of sites available for instantaneous and kinetic sorption); *ω*, the mass transfer coefficient (calculated as $\omega =\frac{\alpha \left(1-\beta \right)RL}{V}$, where α is the first-order kinetic rate coefficient (h^−1^) and L is the column length (cm)); pore water velocity was calculated as $\frac{q}{\theta }$ (where *q* is the water flow rate and *ϴ* is the volumetric water content); *r*^2^, coefficient of determination.

**Table 6:** Simulation results for the transport of Cr(VI) in the loamy sand soil (uniform model).

Simulation Outputs	Flow Rate (cm h^−1^)	CMC-nZVI Suspensions			
		CMC-nZVI 1	CMC-nZVI 2	CMC-nZVI 3	CMC-nZVI 4
Time for solute arrival (h)	2.0	3.75	3.50	1.75	1.75
Time for maximum RC (h)	2.0	29.75	27.75	16.25	15.25
Time for RC < 0.01 (h)	2.0	56.0	48.0	40.0	40.0
Max. Cr(VI) concentration (mg L^−1^)	2.0	99.8	99.8	99.9	99.9
Amount of Cr(VI) removed (mg)	2.0	27.4	27.4	27.5	27.6
Removal efficiency of Cr(VI) (%)	2.0	70.1	70.1	70.3	70.6
Time for solute arrival (h)	4.0	2.00	1.75	1.00	1.00
Time for maximum RC (h)	4.0	15.00	14.50	8.00	7.50
Time for RC < 0.01 (h)	4.0	28.0	26.0	21.0	20.0
Max. Cr(VI) concentration (mg L^−1^)	4.0	99.8	99.9	100.0	100.0
Amount of Cr(VI) removed (mg)	4.0	28.3	28.3	28.4	28.5
Removal efficiency of Cr(VI) (%)	4.0	72.4	72.4	72.6	72.9

CMC-nZVI 1, pH 5 and 0.01 M; CMC-nZVI 2, pH 5 and 0.001 M; CMC-nZVI 3, pH 7 and 0.01 M; and CMC-nZVI 4, pH 7 and 0.001 M; RC, relative concentration.

**Table 7:** Simulation results for the transport of Cr(VI) in the loamy sand soil (two-site sorption model).

Simulation Outputs	Flow Rate (cm h^−1^)	CMC-nZVI Suspensions			
		CMC-nZVI 1	CMC-nZVI 2	CMC-nZVI 3	CMC-nZVI 4
Time for solute arrival (h)	2.0	1.75	2.25	1.75	1.75
Time for maximum RC (h)	2.0	29.75	29.75	28.00	15.25
Time for RC < 0.01 (h)	2.0	80.0	80.0	48.00	44.0
Max. Cr(VI) concentration (mg L^−1^)	2.0	92.5	94.8	99.6	100.0
Amount of Cr(VI) removed (mg)	2.0	27.2	27.4	27.4	27.5
Removal efficiency of Cr(VI) (%)	2.0	69.5	70.1	70.1	70.3
Time for solute arrival (h)	4.0	1.00	1.00	1.00	1.00
Time for maximum RC (h)	4.0	15.00	15.00	13.50	7.50
Time for RC < 0.01 (h)	4.0	52.0	46.0	22.0	21.0
Max. Cr(VI) concentration (mg L^−1^)	4.0	91.0	93.5	99.4	100.0
Amount of Cr(VI) removed (mg)	4.0	28.2	28.3	28.3	28.4
Removal efficiency of Cr(VI) (%)	4.0	72.1	72.4	72.4	72.6

CMC-nZVI 1, pH 5 and 0.01 M; CMC-nZVI 2, pH 5 and 0.001 M; CMC-nZVI 3, pH 7 and 0.01 M; and CMC-nZVI 4, pH 7 and 0.001 M; RC, relative concentration.

## Data Availability

The data analyzed during the current study are available from the corresponding author on reasonable request.

## Appendix A. Calculations of the of Ionic Strength of the Background Electrolyte Solution, the Debye–Huckel Length, and the DLVO Interactions of the CMC-nZVI Nanoparticles

Calculation of the ionic strength (*I*) of the electrolyte solution and the Debye–Huckel length (*k*^−1^) were carried out according to Equations (A1) and (A2), respectively.

(A1) $$I=\frac{1}{2}\overset{n}{\underset{J=1}{\sum }}C_{j}{Z_{j}}^{2}$$

(A2) $$k^{-1}=\sqrt{\frac{e_{0}\varepsilon K_{B}T}{2N_{A}e^{2}I}}$$

The total interaction energy between nanoaggregates (i.e., CMC-nZVI-CMC-nZVI) is assumed to be created from a sphere–sphere interaction. The calculation was carried out according to Bhattacharjee and Elimelech [54]. The electrostatic double layer force (EDL) was calculated as

(A3) $$\Phi_{EDL\;\left(CMC-nZVI-CMC-nZVI\right)}=\frac{2\pi a_{P1}a_{P2}n_{\infty }k_{B}T}{\left(a_{P1}+a_{P2}\right)k^{2}}\left[2\psi_{p1}\psi_{p2}\ln\left(\frac{1+e^{-ky}}{1-e^{-ky}}\right)+\left(\psi_{p1}^{2}+\psi_{p2}^{2}\right)\ln\left(1-e^{-2ky}\right)\right]$$

The attractive van der Waals interaction energies was calculated according to Gregory [55], as follows:

(A4) $$\Phi_{VDW\;\left(CMC-nZVI-CMC-nZVI\right)}=-\frac{A_{121}a_{P1}a_{P2}}{6y\left(a_{P1}+a_{P2}\right)}\left[1-\frac{5.32y}{\lambda }ln\left(1+\frac{\lambda }{5.32y}\right)\right]$$

A list of all symbols and parameters used in Equations (A1)–(A4) are listed in Table A1.

**Table A1:** A list of all symbols and parameters used for the calculations of ionic strength, the Debye–Huckel length, and the total interaction energy between CMC-nZVI nanoparticles.

Symbol	Definition	Units/Comments
*C_j_*	Molar concentration	Mole∙L^−1^
*Z*_j_	Valence of ion	-
*e_0_*	Permittivity of free space	F m^−1^
ɛ	Dielectric Constant	80 for water
*k_B_*	Boltzmann constant	J K^−1^
*T*	Absolute temperature	°K
*N_A_*	Avogadro’s Number	1 mole^−1^
*e*	Electron charge	Coulomb
*I*	Ionic strength	Mole∙m^−3^
$a_{p1}$	The radius of the first interacting CMC-nZVI	nm
$a_{p2}$	The radius of the second interacting CMC-nZVI	nm
$n_{\infty }$	The bulk number density of ions	-
k	The inverse Debye–Huckel length	m
$\psi_{p1}$ $,\;\psi_{p2}$	The surface potentials of interacting CMC-nZVI	mV, Zeta potential value is used
y	The separation distance between two particles	nm
$A_{121}$	The Hamaker constant for: CMC-nZVI-water-CMC-nZVI	3.5 × 10^−20^ J
λ	The characteristics wavelength of the interaction	100 nm (Gregory, 1981 [55])
